# Geometric Morphometric Analysis of Mandibular Symphysis Growth between 12 and 15 Years of Age in Class II Malocclusion Subjects

**DOI:** 10.3390/life13020543

**Published:** 2023-02-15

**Authors:** Ferdinando Ruiz, Pietro Venezia, Vincenzo Ronsivalle, Calogero Lacagnina, Cristina Conforte, Gaetano Isola, Rosalia Leonardi, Antonino Lo Giudice

**Affiliations:** 1 Department of Medical-Surgical Specialties—Section of Orthodontics, School of Dentistry, University of Catania, Policlinico Universitario “G. Rodolico-San Marco”, Via Santa Sofia 78, 95123 Catania, Italy; 2 Department of Medical-Surgical Specialties—Section of Periodontology, School of Dentistry, University of Catania, Policlinico Universitario “G. Rodolico-San Marco”, Via Santa Sofia 78, 95123 Catania, Italy

**Keywords:** geometric morphometrics, orthodontics, mandibular symphysis, Procrustes superimposition, orthodontic treatment, class II malocclusion

## Abstract

The aim of the present paper was to evaluate the morphology changes of the mandibular symphysis (MS) in a longitudinal retrospective cohort of class II untreated subjects. The study sample included 120 subjects followed during normal growth and examined at the age of 12 (T0) and 15 (T1) years. MS was traced using two landmarks and ten sliding semi-landmarks. The acquired morphological data were processed via Procrustes superimposition that allowed to study variation and covariation in MS’form according to specific variables such as age, gender, and skeletal pattern. The first two principal components (PCs) described more than 90 % of the total morphological variation. Both types of form changes of the symphysis could be associated with the different skeletal vertical growth patterns. Age and sex did not interfere with the form of chin symphysis. Moreover, there was no significant covariation between initial MS morphology and form modifications. Clinicians should not expect to be faced with spontaneous changes of the form of the symphysis during the orthodontic treatment of adolescents.

## 1. Introduction

The characteristics and morphology of the mandibular symphysis (MS) significantly impact the orthodontic diagnosis and treatment plan strategies. In particular, MS represents a significant anatomical reference for the facial aesthetic analysis and is also one of the predictors for the direction of the rotational growth pattern of the mandible [1]. Since the form of the MS affects the amount of trabecular bone supporting the lower incisors, it provides valuable information about the anterior lower limit of the dentition during the application of biomechanics for the correction of crowding or dento-skeletal malocclusions. The excessive retraction or proclination of the anterior teeth may generate iatrogenic side effects, such as alveolar bone loss, dehiscence, fenestration, gingival recession, and root resorption [2]. Thus, the analysis of the form of the alveolar bone at the MS region would help define the therapeutic limits for orthodontic tooth movement, especially in class II subjects.

Both genetic and functional factors can influence the morphology of the MS, the latter expressed as the adaptive response to the biomechanical loads developed during the masticatory cycle [3]. In this regard, cortical bone responds to the functional loading in two manners: (1) directly, by the direct muscles attached to the mandible and (2) indirectly, by the forces generated by the muscles to the articulating surfaces of the dentition and condyles [4]. Since there is a relationship between muscle activity and vertical skeletal patterns, the vertical growth direction may indirectly affect the form of MS [5].

Some studies have dealt with the morphological characteristics of the mandibular symphysis and the skeletal pattern [6,7,8]; however, most of these studies, except for one [9], were limited to conventional cephalometric or linear measurements that do not precisely reproduce the morphology of the curvature of the MS.

Recently, geometric morphometric (GMM) analysis has become an important scientific tool in orthodontics to investigate modifications in skeletal morphology that can explain complex morphology differences more successfully than coefficients from the traditional morphometric analysis [10]. In this regard, the present study aimed to use GMM to evaluate the form of MS from 12 to 15 years, which generally represents the age range associated with the orthodontic treatment, by longitudinally assessing a retrospective sample of untreated class II subjects.

## 2. Materials and Methods

### 2.1. Study Sample

The present retrospective study was performed, including lateral cephalograms from the American Association of Orthodontists Foundation (AAOF) Craniofacial Growth Legacy Collection (www.aaoflegacycollection.org). This archive consists of nine known collections of longitudinal craniofacial growth records in the United States and Canada, including cephalograms taken each year in children who never received orthodontic treatment.

Firstly, we searched for male and female subjects who had taken lateral cephalograms at 12 ± 6 months (T0) and 15 ± 6 months (T1), respectively. According to these preliminary criteria, 227 subjects were recruited from the Burlington, Fels, Iowa, and Oregon growth studies databases. Afterward, all radiographs were examined and the study sample was defined according to the following criteria: (1) skeletal class II malocclusion, with ANBˆ (>4°); (2) absence of fixed appliances, including space maintainers; (3) teeth in occlusion; (4) absence of extreme craniofacial pattern. The study sample consisted of 120 subjects (64 females and 56 males) and 240 cephalograms (T0 = 12 years old, T1 = 15 years old).

Since the cephalograms included in the present study were retrieved from different radiographic devices, a preliminary image calibration was performed to exclude the magnification error from the morphometric analysis. For each cephalogram, the magnification correction was carried out by placing two points on the calibrated ruler at a distance of 20 mm (View box 4.1 Dhal software, Kifissia, Greece).

Finally, the FMAˆ was used as a cephalometric parameter for distinguishing vertical growth patterns and generating 3 subgroups according to the respective diagnosis: 46 normodivergent (FMAˆ ≥ 26°), 43 hypodivergent (FMAˆ = 25° ± 1°), and 31 hyperdivergent subjects (FMAˆ ≤ 24°).

### 2.2. Definition of the Curve of the Mandibular Symphysis

**Figure 1:**
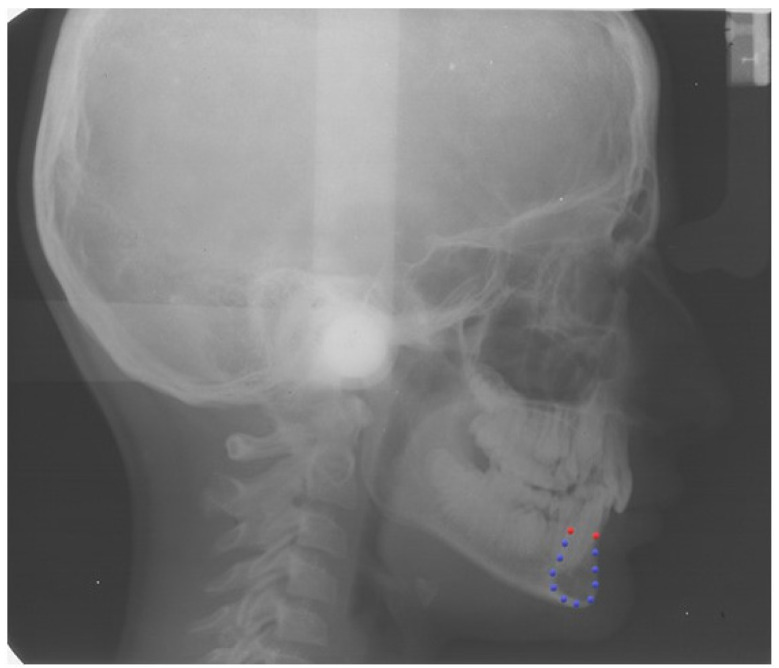
Example of a longitudinal cephalogram of recruited patient from the American Association of Orthodontists Foundation (AAOF) Craniofacial Growth Legacy Collection. Red dots = fixed landmarks (VS point and LS point); blue dots = semi-landmarks.

A single continuous curve was digitized following the outline of the symphysis on each lateral cephalogram to assess the general outline (View box 4.1 Dhal software, Kifissia, Greece). As geometric morphometrics does not allow the use of curves, we placed twelve landmarks on the curve of the symphysis. Firstly, two points were identified at the vestibular superior (VS point) and lingual superior (LS point) margins of the cortical bone of the symphysis. Since these two points derived from the specific anatomy of the symphysis and were easily identifiable in each specimen, they were considered homologous and used as fixed points or landmarks. The remaining ten points were randomly placed along the curve, then distributed uniformly using a specific feature of the software and allowed to slide along the curve to minimize the bending energy as semi-landmarks [11] (Supplementary Table S1). By repeating this process 5 times iteratively, we reached a position that was considered homologous between all the specimens and that allowed us to consider the 10 semi-landmarks as fixed points (Figure 1) [12].

### 2.3. Procrustes Superimposition

All the coordinates were exported in three different datasets containing, respectively, the coordinates of the patients at T0, at T1, and data acquired at T0 and T1 together. Each dataset was imported into a specific software for geometric morphometric analysis (MorphoJ, Version 2.0, Klingenberg lab, The University of Manchester) [13].

A Procrustes fit was performed for each dataset and the points were superimposed by generalized Procrustes alignment; the obtained Procrustes coordinates were projected in Kendall tangent space. Univariate and multivariate normality was evaluated using MVN (http://www.biosoft.hacettepe.edu.tr/MVN/, Ver. 1.6); particularly, the Shapiro–Wilk test, the Cramer–von Mises test, the Lilliefors test, the Mardia test, and the Royston test were performed [14].

### 2.4. Statistical Analysis

The Procrustes coordinates of the T0 and T1 datasets were used to perform the principal components analysis (PCA) to obtain the principal components that described the major features of MS change in each group [15]. The same analysis was also used, including both T0 and T1 datasets, to increase the sensitivity of the analysis. The discriminant function analysis (DFA) was performed to evaluate the influence of gender and age in MS’ form change at both T0 and T1. At the same time, a T-square test and a permutation test were also executed to obtain a *p*-value [16]. The canonical variation analysis was also carried to evaluate the influence of biotype in morphological change at T0 and T1, analyzing the group structure in multivariate data [17].

The third dataset, including both T0 and T1 coordinates, was analyzed using the DFA test to evaluate the magnitude of age in form change. Two different analyses were performed: the first analysis compared the coordinates at 12 years and 15 years and the second analysis compared the same coordinates for each biotype. Permutation tests were executed and a *p*-value was obtained.

Finally, the two-block partial least squares analysis (2B-PLS) was performed for the whole sample to assess the amount of covariation between the initial position of each subject (symphysis) and the change of form during growth, i.e., between T0 (12 years) and T1 (15 years) [18]. The analysis was performed with MorphoJ software (Version 2.0, Klingenberg lab, The University of Manchester) and the RV coefficient evaluated the covariation strength [19]. The alpha level for all statistical analyses was set at 0.05.

Intra-operator and inter-operator reliability were carried out 1 month after 30 randomly selected cephalograms to evaluate the accuracy of tracing and landmark placement. Concerning cephalogram tracing, the intraclass correlation coefficients test (ICC) showed values above 0.95 and above 0.89 for both intra- and inter-reliability tests. Concerning landmark placement, the ICCs also showed high reliability, with 0.999 for x coordinates and 0.987 for y coordinates.

## 3. Results

### 3.1. Generalized Procrustes Superimposition and PCA

Table 1 shows the demographics and variable used in PCA. With Procrustes analysis, all the specimens were superimposed and all the information regarding rotation, translation, and volume among the observations were uniformed to perform principal components analysis. Due to Procrustes alignment, four degrees of freedom were lost in each dataset. For the T0 dataset (12 Years), the number of significant principal components was assessed at 7 (Pc1–Pc7) and accounted for 98.903% of the cumulative variation. For the T1 dataset (15 Years), the number of significant principal components was also 7 (Pc1–Pc7) and accounted for 98.890% of the overall variation. For both T0 and T1, almost 90% of the cumulative variance was described by the first two PCs (Table 1).

**Figure 2:**
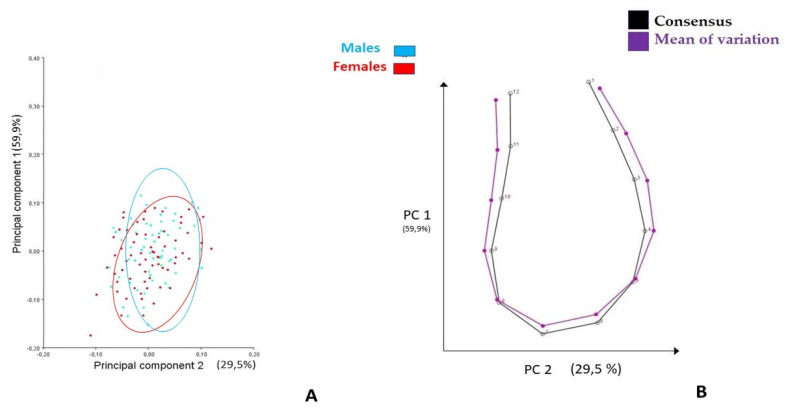
First two PCs describing the modifications of mandibular symphysis at T0 (12 Y-O age), according to Procrustes superimposition and PCA. (**A**) Pc scores clustered by gender; (**B**) graphical illustration; black curve represents consensus, purple curve represents the mean of variation.

**Figure 3:**
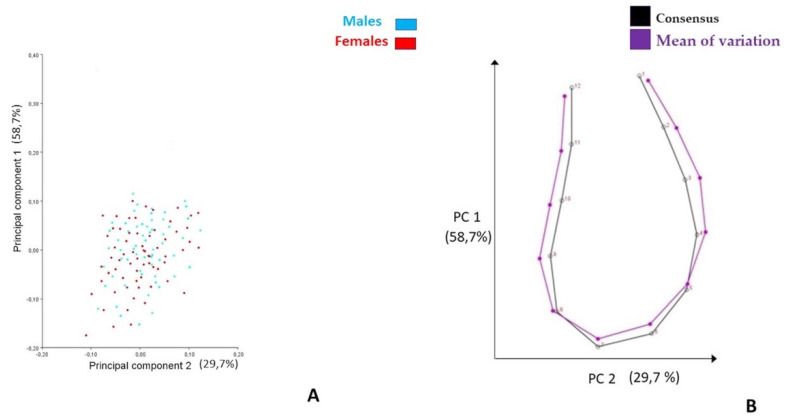
First two PCs describing the modifications of mandibular symphysis at T1 (15 Y-O age), according to Procrustes superimposition and PCA. (**A)** Pc scores clustered by gender; (**B**) graphical illustration; black curve represents consensus, purple curve represents the mean of variation.

PC 1 described the Ms’form change along the axial projection, while PC 2 described it along the sagittal axis; both types of modifications of the symphysis could be associated with the different morphological characteristics related to skeletal vertical growth patterns (Figure 2 and Figure 3, Table 2). A similar description of the form change was obtained, including T0 and T1 datasets in the PCA (Supplementary Figures S1–S3).

### 3.2. Age, Sex, and Form Correlation

According to the discriminant function analysis, there was not a statistically significant difference between males and females in both the T0 (*p*-value: 0.56) and the T1 dataset (*p*-value: 0.11). Similarly, age did not influence the form of chin symphysis both at T0 and T1 (*p*-value: 0.71) (Table 3). Using the canonical variation analysis, a statistically significant difference in Ms’form was found between hyperdivergent and hypodivergent patterns in both T0 and T1 datasets (T0 *p*-value: <0.001; T1 *p*-value: <0.001). Meanwhile, there were no statistically significant differences between other combinations of skeletal patterns (normo/hyper: T0 *p*-value: 0.062 T1 *p*-value: 0.073; normo/hypo T0 *p*-value: 0.34–T1 *p*-value: 0.38) (Table 4).

### 3.3. Form Covariation and Inter-Timing Assessment of Morphological Change

To evaluate the covariation between the morphology of the mandibular symphysis at 12 years and at 15 years, a two-block partial least squares analysis (2B-PLS) was performed using T0 as the reference group and T1 as the comparison group. Both groups were pooled according to the vertical skeletal pattern. According to the 2B-PLS, form change did not show significant covariation with initial form (RV coefficient: 0.486, *p* value < 0.001). PLS1 accounted for 87.01 per cent of the total covariance, while PLS2 accounted for 7.57 per cent of the total covariance. Since growth did not interfere with the form changes, as suggested by the superimposition of the mean morphological changes at T0 and T1, the vector described by the PLS1 would reflect the mean morphological variation expressed in the principal components analysis and be related to the sagittal and vertical intragroup form characteristics attributed to different facial biotypes. It should be underlined that the smaller sample size may have contributed to the inflation of the RV value (Table 5).

**Figure 4:**
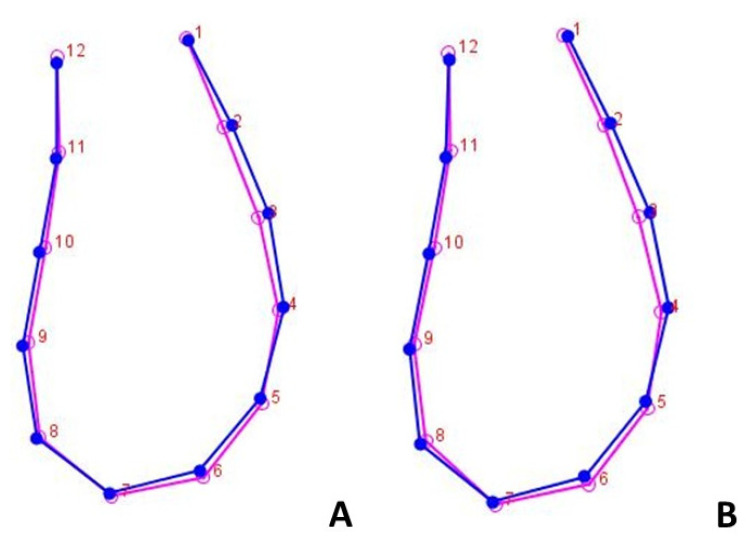
Superimposition of the curves of mandibular symphysis between males and females according to the discriminant function analysis. (**A**) T0 = 12 years; (**B**) T1= 15 years. Blue-colored curve represents male subjects; pink-colored curve represents female subjects.

**Figure 5:**
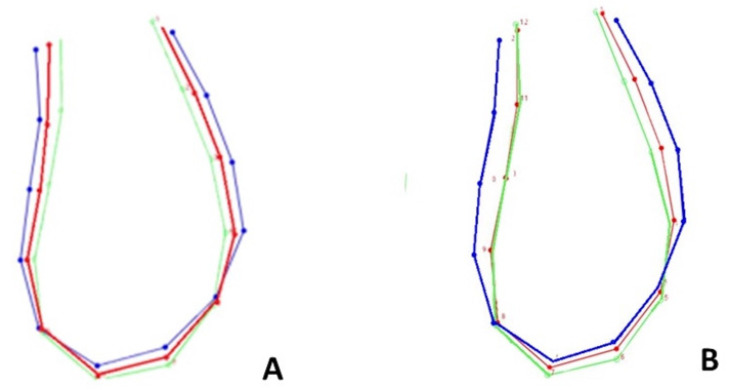
Superimposition of the curves of mandibular symphysis between different skeletal patterns according to the discriminant function analysis. (**A**) T0 = 12 years; (**B**) T1 = 15 years. Blue-colored curve represents hypodivergent subjects; red-colored curve represents normodivergent subjects; green-colored curve represents hyperdivergent subjects.

**Figure 6:**
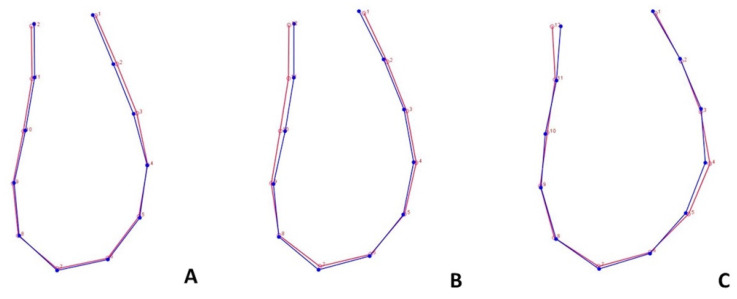
Superimposition of the curves of mandibular symphysis between different skeletal patterns according to the discriminant function analysis. (**A**) Hyperdivergent subjects; (**B**) normodivergent subjects; (**C**) hypodivergent subjects. Blue-colored curve represents patients at T0; red-colored curve represents patients at T1.

The discriminant function analysis (DFA) showed no statistically significant differences between different observational timing according to gender (Figure 4) and facial biotype variables (Figure 5 and Figure 6), confirming data acquired from the two-block partial least squares analysis (2B-PLS).

## 4. Discussion

The morphology of mandibular symphysis is an essential parameter in the orthodontic treatment plan, especially when dentoalveolar compensation is required to camouflage for the underlying sagittal dento-skeletal discrepancy [20]. The cortical bone of the symphysis is thinner than the other mandibular parts. It represents a critical limitation when planning the amount of lower incisors’ proclination, retraction, and corono-radicular torque [21]. Since the anteroposterior movement of the incisors can influence the morphology of the symphysis as well as the risk for iatrogenic injuries, such as gingival recession or dehiscence [22], it is crucial to identify the predictive factors that could be correlated with the morphology of MS [23]; this could help clinicians in establishing the appropriate treatment strategies in the choice for teeth extraction, incisor proclination, or stripping [24]. In this regard, previous studies showed that there is a significant relationship between facial biotype, skeletal malocclusion, and alveolar bone thickness/height [25]. The evidence would suggest that the vertical skeletal/facial pattern has a significant influence on the morphologic variation of the mandibular symphysis [26,27]. In addition, a recent study investigating the morphology of the mandibular symphysis in Class III subjects would confirm the relation between the skeletal pattern and the morphology of the symphysis [28]. However, these studies did not evaluate how the form of the symphysis may change during growth and whether this change is related to the initial craniofacial pattern. This could be critical from the clinical perspective, since orthodontic treatment is generally performed during adolescence when some potential growth modification of the mandible can still occur [29]. To the best of our knowledge, this is the first study in the literature investigating the form changes of the mandibular symphysis during growth, particularly in class II subjects between the ages of 12 and 15 years old, representing the usual age range of orthodontic treatment.

Two approaches are documented in the pertinent literature for the study of mandibular symphysis. Following Klingenberg’s work [30], it is possible to use a homology-free approach that does not envisage semi-landmarks but exploits the surrounding anatomical structures to identify the points used in the analysis [28]. It is also possible to take advantage of a more traditional approach that involves the use of semi-landmarks [9]. It could be argued that the usage of semi-landmarks is a controversial procedure in morphometric studies. As previously suggested, semi-landmarks do not always allow to comprehensive identify a specific anatomical morphology, since they exclude potential characteristics developed ex novo during the evolution of the species. However, considering the limited observational timing of the present study, it is unlikely that the mandibular symphysis may have shown MS’form changes due to ex novo occurrences of morphological features. In this regard, both methodologies have been previously used to investigate symphysis morphology [9,28].

### 4.1. Generalized Procrustes Superimposition and PCA

The largest PCs (PC1 and PC 2), which accounted for almost 90% of the cumulative variance, expressed the form variation along the vertical and sagittal craniofacial axes and generally attributed to the different skeletal vertical growth pattern, i.e., hyperdivergent vs. hypodivergent subjects [30]. In particular, subjects within the hyperdivergent subgroup showed a narrower and elongated symphysis compared with the hypodivergent subgroup. This is in agreement with previous quantitative findings [31,32] and would suggest that hyperdivergent individuals can be more exposed to iatrogenic injuries such as gingival recession or dehiscence, in particular for the treatment of mandibular crowding and for the dentoalveolar compensation of class II using intermaxillary elastics or auxiliaries. As a consequence, these patients are more likely to be treated with orthodontic extraction to correct the arch length discrepancy or the sagittal discrepancy [33,34]. This would confirm the importance of establishing an appropriate treatment plan based on an evaluation of the morphology of the mandibular symphyses. Since we included only subjects with class II malocclusion, the morphometric analysis performed in this study did not consider the general influence of the sagittal skeletal pattern in the morphology of mandibular symphysis.

### 4.2. Age, Sex, and Ms’Form Correlation

When evaluating the morphology of the mandibular symphysis according to gender, no differences were found between included subjects. Nevertheless, previous evidence suggested that males exhibit larger symphysis dimensions than females. Sexual dimorphism has been reported between males and females about different aspects of the craniofacial complex [35]. The contrast between the present findings and those from the previous study can be explained considering that Procrustes superimposition eliminates any information about dimensional data, since each individual (landmark configuration) is translated to a common origin and scaled to a unit centroid size. Thus, it may be assumed that no differences in the morphology of the symphysis should be expected between males and females, while males can exhibit more significant vertical and sagittal bone availability compared with females [36].

Instead, data obtained comparing different facial biotypes showed statistically significant differences between hypodivergent and hyperdivergent subjects, corroborating previous assumptions that the vertical skeletal/facial pattern could significantly influence the morphologic variation of the mandibular symphysis [26]. Instead, no differences were detected between normodivergent/hypodivergent subjects and normodivergent/hyperdivergent subjects; however, it could be possible that the limited sample size allowed the identification of significant differences only among hypodivergent and hyperdivergent subjects that represent the two extremes of the skeletal growth patterns. In this regard, only further studies with a greater sample size could elucidate this assumption.

### 4.3. Ms’Form Covariation and Inter-Timing Assessment of Morphological Change

The RV value of 48.60% showed no significant covariation between the initial form of the symphysis and the anatomical change associated with residual growth. Accordingly, clinicians should not expect to face spontaneous changes in the symphysis morphology during the orthodontic treatment of adolescents. This could be important for hyperdivergent subjects since they are generally more exposed to the iatrogenic effects occurring during the orthodontic treatment, such as the risk for dehiscence or fenestration of the lower incisors.

Although the findings of the present study are limited to a small sample size, they would confirm the importance of integrating the evaluation of the form and morphology of the symphysis with skeletal and facial parameters not only to identify the patterns of skeletal relationships (including the prediction of size and direction of the mandible in growing subjects) but also for the establishment of the appropriate biomechanics finalized to the most balanced functional and aesthetic outcomes.

### 4.4. Limitations

The sample characteristics were limited to the subjects retrieved from the AAOF Craniofacial Growth Legacy Collection that fulfilled our inclusion and exclusion criteria. The sample consisted of normal untreated subjects of White–Caucasian race who lived around the 1950s and exhibited various craniofacial morphologies. The historical nature of the data might not accurately reflect the trends of a contemporary population. In addition, the subjects were pooled from different geographical regions with potentially different environmental conditions, nutritional habits, genetic ancestry, and radiographic sources, all of which might reflect on craniofacial morphology and growth.

CBCT can provide a higher definition of the morphology of the symphysis, in particular at the outer and inner cortical level, compared with the conventional L-L radiographs retrieved for this study. However, such limitation should not be considered a major concern, since the identification of the characteristics of the symphysis from L-L is consistent with the clinical usage of conventional 2D radiographs for orthodontic diagnoses and treatment plans according to the A.L.A.R.A. and A.L.A.D.A. principle [37,38].

## 5. Conclusions

The morphological changes of the symphysis could be associated with the different morphological characteristics related to skeletal growth patterns. In particular, the vertical growth pattern would influence the form of the mandibular symphysis. These differences can be clinically favorable or unfavorable according to the orthodontic biomechanics applied.

Age and sex did not interfere with the morphology of chin symphysis.

Clinicians should not expect to face spontaneous changes of MS’form during the orthodontic treatment of adolescents.

## Figures and Tables

**Table 1:** Demographics table summarizing the features of the sample and the variables used in principal components analysis.

Sample Characteristics	N° of Observations
**Gender**	
Female Male	64 56
**Age**	
12 Years Old (T0)	120 *
15 Tears Old (T1)	120 *
**Vertical Skeletal Pattern**	
Normodivergent (FMAˆ ≥ 26°)	46
Hypodivergent (FMAˆ = 25° ± 1°)	43
Hyperdivergent subjects (FMAˆ ≤ 24°)	31

* Each patient underwent 2 X-rays.

**Table 2:** Principal components (PC) and percentage of variance of total morphological changes at both T1 and T0.

T1 PCs	% Variance	Cumulative %
PC 1.	60.897	60.897
PC 2.	21.292	82.189
PC 3.	6.619	88.808
PC 4.	6.176	94.984
PC 5.	2.198	97.183
PC 6.	1.001	98.184
PC 7.	0.627	98.890
**T0** **PCs**	**% Variance**	**Cumulative %**
PC 1.	59.223	59.223
PC 2.	24.123	83.346
PC 3.	6.99	90.145
PC 4.	5.84	95.829
PC 5.	1.551	97.381
PC 6.	0.933	98.314
PC 7.	0.589	98.903

**Table 3:** Discriminant function analysis (DFA) performed to evaluate the influence of gender and age in MS’form change at both T0 and T1.

Variable	T0	T1
Gender	*p*-value: 0.56	*p*-value: 0.11
Age	*p*-value: 0.71	

**Table 4:** Canonical variation analysis (CVA) performed to investigate the influence of the skeletal pattern. *p*-value based on permutation test. Reference group = normodivergent, comparison groups = hyperdivergent and hypodivergent.

Variable	T0	T1
Skeletal pattern		
- Hyper/Hypo - Normo/Hyper - Normo/Hypo	*p*-value: <0.001 *p*-value: 0.062 *p*-value: 0.34	*p*-value: <0.001 *p*-value: 0.073 *p*-value: 0.38

**Table 5:** Assessment of the amount of covariation between the initial position of the symphysis and the change of MS’ morphology during growth (T0-T1). RV (correlation coefficient) and *p* value based on two-block partial least squares analysis (2B-PLS). Reference group = T0 patients, comparison group = T1 patients; both groups pooled by vertical skeletal pattern.

2B-PLS	RV: 0,486; *p*-Value: < 0.001	Cumulative Covariance
PLS1	87.01%	87.01%
PLS2	7.57%	94.58%

## Data Availability

Data are available upon reasonable request to the corresponding author.

## Supplementary Materials

The following supporting information can be downloaded at: https://www.mdpi.com/article/10.3390/life13020543/s1, Figure S1: PCA plot for T0 and T1 data clustered by gender; Figure S2: PC coefficients for each point coordinates in T0 + T1 dataset; Figure S3: Graphical visualization of the distribution of the eigenvalues and their PCs for T0 + T1 dataset; Table S1: Description and classification of landmarks and semi-landmarks placed along the symphysis outline.
