# Supplementary material for: Geometric Morphometric Analysis of Mandibular Symphysis Growth between 12 and 15 Years of Age in Class II Malocclusion Subjects
Source: Life (Basel). 2023 Feb 15;13(2):543. doi: 10.3390/life13020543 (PMC9959364; doi:10.3390/life13020543)
Supplement: Supplementary file 1 [file life-13-00543-s001.zip › life-2008210-supplementary.pdf]

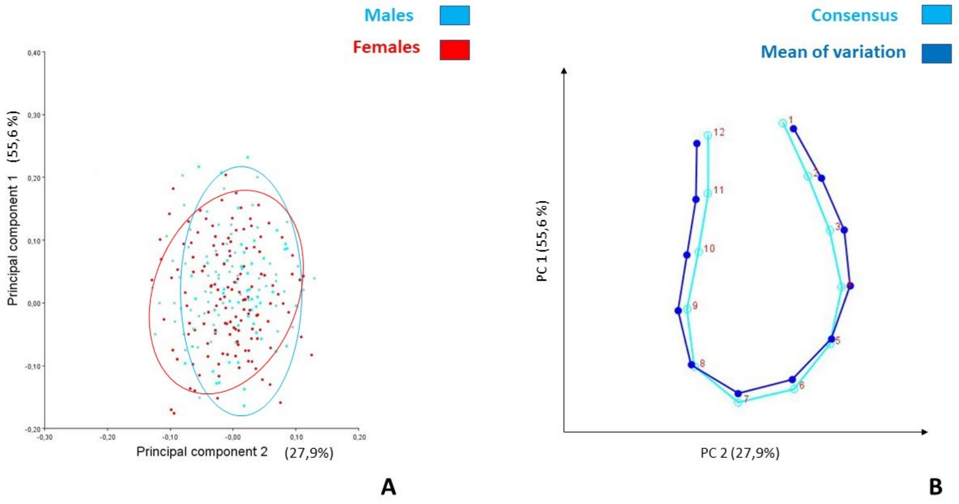

Supplementary Figure S1: PCA plot for T0 and T1 data clustered by gender.

Principal Component Coefficients

	PC1	PC2	PC3	PC4	PC5	PC6	PC7
x1	-0,277237	0,128503	-0,462328	-0,055266	0,330703	0,270817	0,333246
y1	0,153606	-0,182759	-0,215702	-0,025173	0,256450	0,099331	0,236422
x2	-0,359491	-0,014181	-0,157693	-0,261472	0,063339	-0,325189	-0,404601
y2	0,057599	-0,219293	-0,161660	-0,147132	0,122956	-0,175328	-0,194532
x3	-0,376412	-0,116895	0,265398	-0,201473	-0,437224	-0,145213	0,024265
y3	-0,006929	-0,240888	-0,046947	-0,138776	-0,102035	-0,108543	-0,061559
x4	-0,233801	-0,047941	0,493739	0,128375	-0,005360	0,386336	0,242747
y4	-0,040230	-0,211396	-0,010551	-0,089077	-0,028773	0,055616	0,065015
x5	-0,040809	0,091571	0,162474	0,467615	0,298213	-0,006909	-0,305053
y5	-0,130510	-0,196389	-0,020944	-0,156789	-0,072249	0,149750	0,145202
x6	0,040387	0,127589	-0,163770	0,253633	-0,044335	-0,251679	0,087478
y6	-0,251380	-0,166271	0,132118	0,019367	0,184465	0,262165	-0,129260
x7	-0,002042	0,104984	-0,203325	0,137389	-0,101025	-0,084088	0,073920
y7	-0,228347	0,055914	0,024415	0,328046	0,180184	-0,258713	-0,011896
x8	0,082168	0,252856	-0,245623	-0,149583	-0,324279	0,073279	0,164019
y8	-0,048948	0,236109	0,008538	0,064441	-0,122138	-0,111228	0,079035
x9	0,241031	0,209681	0,026515	-0,365155	0,077709	0,356584	-0,357234
y9	0,037345	0,219487	0,054399	0,060217	-0,060967	-0,050003	0,011114
x10	0,307681	-0,004453	0,291845	-0,286192	0,394600	-0,153884	-0,020128
y10	0,088841	0,238517	0,050175	0,037349	-0,146413	0,019841	-0,044227
x11	0,322883	-0,239121	0,207955	-0,038843	0,059990	-0,360864	0,424755
y11	0,149895	0,249285	0,077090	0,007346	-0,104532	0,058435	-0,081358
x12	0,295643	-0,492593	-0,215186	0,370972	-0,312332	0,240811	-0,263415
y12	0,219057	0,217684	0,109068	0,040181	-0,106948	0,058677	-0,013957

Supplementary Figure S2. Eigenvalues coefficients for PCA evaluation.

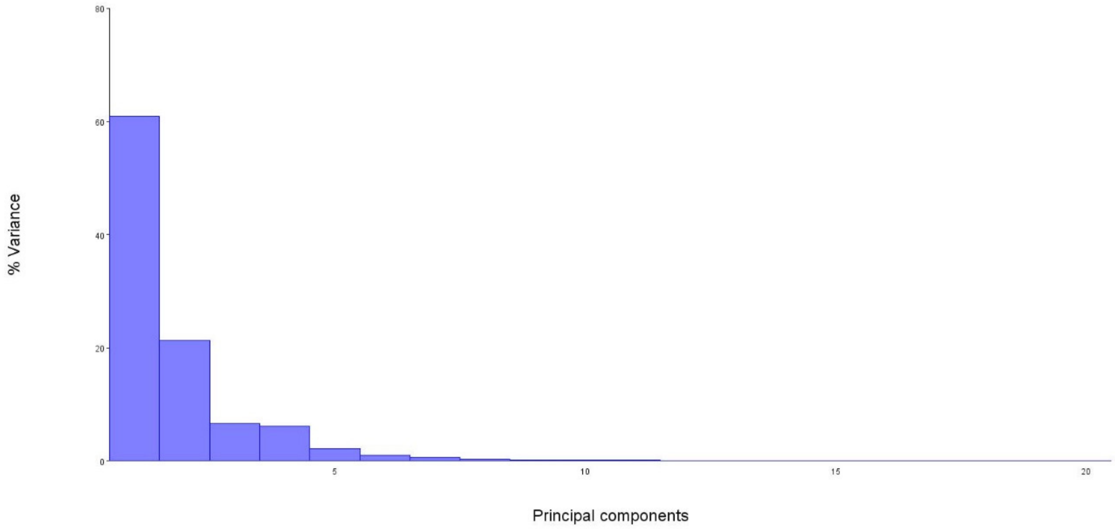

Supplementary Figure S3. Graphical distribution of eigenvalues according to the PCA

Point Name	Point type
Vestibular-superior (VS point)	Fixed Point (type II)
Lingual-superior (LS point)	Fixed Point (type II)
SML – 1	Sliding along curve semi-landmark
SML – 2	Sliding along curve semi-landmark
SML – 3	Sliding along curve semi-landmark
SML – 4	Sliding along curve semi-landmark
SML – 5	Sliding along curve semi-landmark
SML – 6	Sliding along curve semi-landmark
SML – 7	Sliding along curve semi-landmark
SML – 8	Sliding along curve semi-landmark
SML – 9	Sliding along curve semi-landmark
SML – 10	Sliding along curve semi-landmark

Supplementary Table S1. Description and classification of landmarks and semi-landmarks placed along the symphysis outline.
